# Understanding heterogeneity in psychiatric disorders: A method for identifying subtypes and parsing comorbidity

**DOI:** 10.1111/pcn.13829

**Published:** 2025-04-30

**Authors:** Aidas Aglinskas, Alicia Bergeron, Stefano Anzellotti

**Affiliations:** ^1^ Department of Psychology and Neuroscience Boston College Chestnut Hill Massachusetts USA

**Keywords:** disorders, heterogeneity, machine‐learning, neuroimaging

## Abstract

**Aim:**

Most psychiatric and neurodevelopmental disorders are heterogeneous. Neural abnormalities in patients might differ in magnitude and kind, giving rise to distinct subtypes that can be partly overlapping (comorbidity). Identifying disorder‐related individual differences is challenging due to the overwhelming presence of disorder‐unrelated variation shared with healthy controls. Recently, Contrastive Variational Autoencoders (CVAEs) have been shown to separate disorder‐related individual variation from disorder‐unrelated variation. However, it is not known if CVAEs can also satisfy the other key desiderata for psychiatric research: capturing disease subtypes and disentangling comorbidity. In this paper, we compare CVAEs to other methods as a function of hyperparameters, such as model size and training data availability. We also introduce a new architecture for modeling comorbid disorders and test a novel training procedure for CVAEs that improves their reproducibility.

**Methods:**

We use synthetic neuroanatomical MRI data with known ground truth for shared and disorder‐specific effects and study the performance of the CVAE and non‐contrastive baseline models at detecting disorder‐subtypes and disentangling comorbidity in brain images varying along shared and disorder‐specific dimensions.

**Results:**

CVAE models consistently outperformed non‐contrastive alternatives as measured by correlation with disorder‐specific ground truth effects and accuracy of subtype discovery. The CVAE also successfully disentangled neuroanatomical loci of comorbid disorders, due to its novel architecture. Improved training procedure reduced variability in the results by up to 5.5×.

**Conclusion:**

The results showcase how the CVAE can be used as an overall framework in precision psychiatry studies, enabling reliable detection of interpretable neuromarkers, discovering disorder subtypes and disentangling comorbidity.

The heterogeneous nature of many psychiatric and neurodevelopmental disorders can impede the diagnosis of patients and the development of targeted support options.^1^, ^2^, ^3^, ^4^, ^5^ Including neural measures may help identify subtypes that benefit from specific interventions. Recently, there has been an emerging focus on neuroimaging‐based subtyping to identify groups of patients sharing similar disorder‐related neural alterations. This is challenging because genetic and environmental factors also cause brain differences unrelated to psychiatric or neurodevelopmental disorders.^6^ Differences in age, gender, and scan sites further obfuscate disorder‐related neuromarkers. Separating disorder‐specific from shared variation could improve neuromarker discovery and enable targeted interventions.^7^ Methods for studying individual variability should satisfy the following desiderata: capturing graded, categorical and comorbid neural features, and doing so in a reliable and generalizable way.

A new deep‐learning approach—contrastive variational autoencoders (CVAEs)—shows promise in meeting these desiderata^8^: contrastive variational autoencoders (CVAEs). CVAEs can be trained to separate dimensions of variation between brains that are common to all participants from dimensions of variation that are specific to the population of interest. They use two data sets—typical controls and the target population—to learn two feature spaces: one for all participants and one unique to patients. The latter “disorder‐specific” features can then be correlated with symptoms and used for further analyses. Latent features can be linked with clinical symptoms and neural variability to identify biological pathways, predict diagnosis, cluster patients by progression, and distinguish groups likely to respond to similar interventions. CVAE models are rapidly increasing in popularity and have been applied to a diverse set of real neuroimaging datasets spanning different disorders, such as Autism,^9^, ^10^, ^11^ schizophrenia,^12^ Parkinson's^13^ and Alzheimer's.^14^ While these results show that CVAEs can capture gradual individual differences in diverse populations, the remaining desiderata have not been tested, namely: whether CVAEs can capture not only graded (symptom severity) but also categorical (disorder subtype) differences; whether they can do so in the presence of disease comorbidity; and which training methods maximize models' robustness, reliability and generalization capacities.

This paper provides these missing contributions. Using synthetic neuroanatomical data with known ground truth, we show that CVAEs accurately capture both graded disorder severity and distinct subtypes. We introduce a modified CVAE architecture to effectively disentangle disorder comorbidity, identifying interpretable neural loci that are either uniquely or jointly affected by overlapping disorders. Synthetic data provide a controlled environment to rigorously benchmark model performance under conditions that cannot be accurately tested with real‐world datasets which lack a definitive ground truth. In addition, we introduce an ensemble training procedure for CVAEs to enhance robustness across hyperparameters, dataset size, and disorder complexity. Overall, this paper showcases how CVAEs can be used as an overall framework for precision psychiatry to derive precise and interpretable neuromarkers, discover disorder subtypes and disentangle disorder comorbidities in a way that is reliable and generalizable.

## Methods

**Fig. 1:**
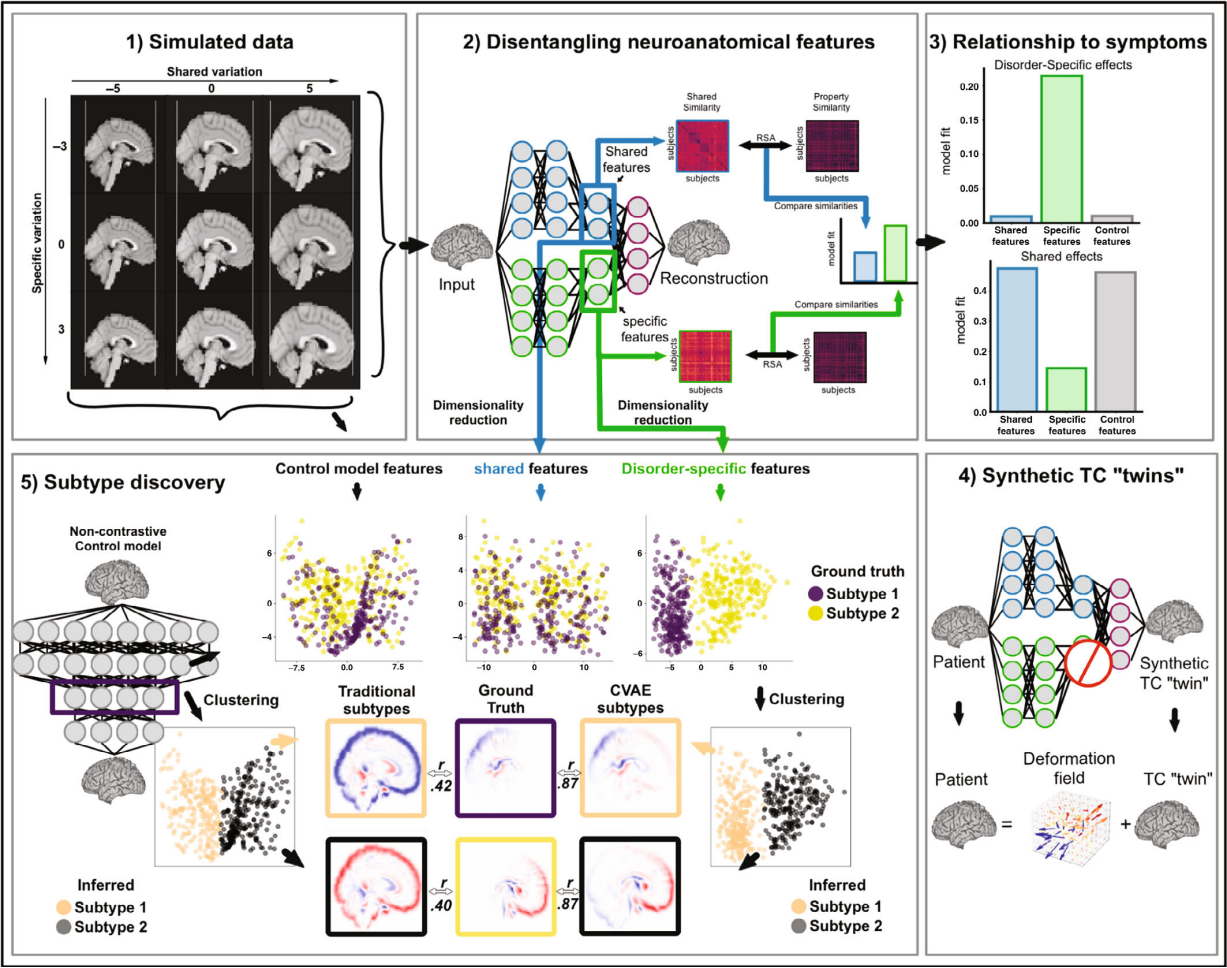
CVAE overview. (1) Simulated data used in the experiment. A template brain was manipulated in two dimensions: shared variation, which either expanded or compressed the whole brain, and disorder‐specific variation, which additionally expanded or compressed specific loci. (2) Disentangling neuroanatomical features. CVAE architecture and analysis overview: models are trained using synthetic data. Once trained, shared and disorder‐specific latent features can be used for downstream analyses, like clustering or RSA. (3) Relationship to symptoms. Using RSA to correlate latent features to ground‐truth measures shows that disorder‐specific features capture variation in ground‐truth symptoms better than shared features or features from baseline VAE models. (4) Synthetic “TC” twins. Schematic of the procedure used to generate counterfactuals matched to individual patients on disorder‐unrelated features. More formally, each brain is reconstructed twice: once using both shared and disorder‐specific features and again using only shared features, with disorder‐specific features set to zero. The difference between reconstructed and TC twin brains can be used to identify disorder‐specific neural loci in individual patients. (5) Subtype discovery. Using CVAE disorder‐specific latent spaces allows us to identify disorder‐subtypes better than non‐contrastive approaches. Scatter plots show that CVAE's disorder‐specific latent features differentiate between disorder subtypes while shared & control model latent features do not (Subtype 1 & 2, ground truth). Clustering in the space of CVAE disorder‐specific latent features reveals more accurate neuroanatomical loci associated with each subtype compared to clustering using the baseline model's features (Subtype 1 & 2, inferred). CVAE inferred subtypes correlate better with ground truth clusters (subtype 1, *r* = 0.87; subtype 2, *r* = 0.87) than subtypes inferred by baseline model (subtype 1, *r* = 0.42; subtype 2, *r* = 0.40).

**Fig. 2:**
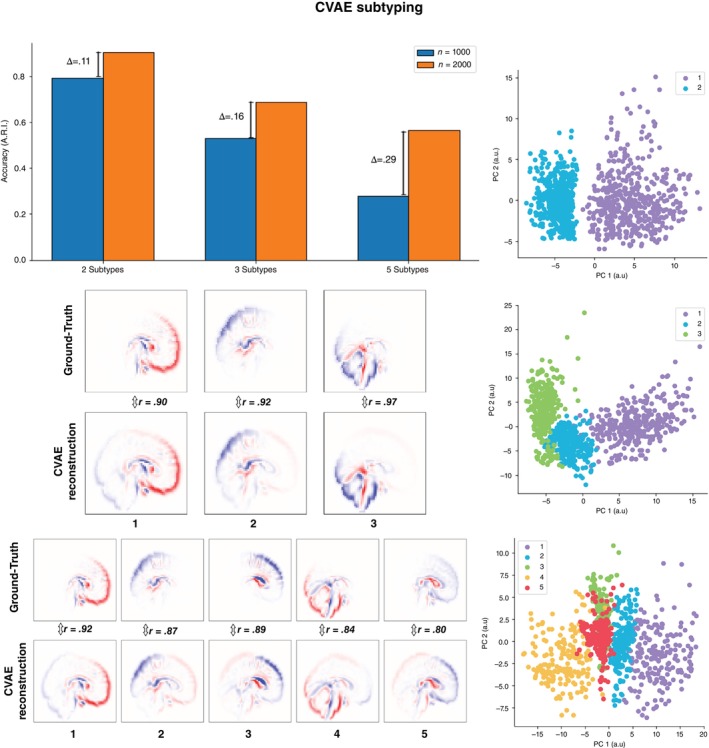
Subtyping neuroanatomy using CVAE. Barplot shows clustering accuracy comparing ground truth and CVAE inferred subtypes (adjusted Rand index, chance level = 0) for a varying number of subjects (1000 or 2000) and a varying number of subtypes (two, three, or five subtypes). Increasing the number of subtypes results in lower accuracy. Increasing the number of subjects improves the subtyping accuracy with increasing improvements as the number of subtypes increases. Scatterplots show subject similarity (*n* = 2000) in a 2D space (dimensions reduced using PCA) for different subtype scenarios (two, three, or five subtypes); colors represent CVAE inferred subtype assignment. Brain plots show ground‐truth neurosubtypes (top rows) and CVAE‐inferred neurosubtypes (bottom rows).

**Fig. 3:**
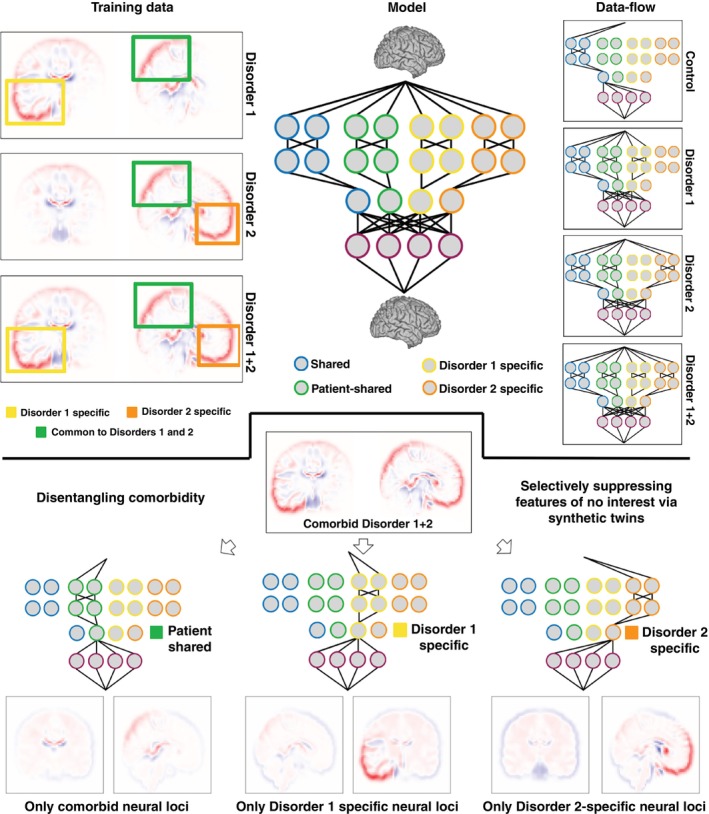
Using CVAEs to model disease comorbidity. Training data: We generated a synthetic dataset consisting of disorder 1, disorder 2, and disorder 1 + 2 subjects. Each disorder had shared (common to all patients and controls), specific (unique to each disorder), and comorbid (common across disorders) deformations. Model: this CVAE model had four distinct feature spaces: shared between all subjects (in common to all controls and all patients), patient‐shared (in common to disorder 1, disorder 2, and disorder 1 + 2 patients), disorder 1 specific (present only in disorder 1 subjects) and disorder 2 specific (present only in disorder 2 subjects). Disentangling comorbidity: We used data from comorbid disorder 1 + 2 subjects, combined with synthetic‐twin approach to selectively identify loci associated with only disorder 1, only disorder 2, and only comorbid loci.

To test CVAE performance with known ground truth, we generated synthetic brains for two groups (“pseudo‐controls” and “pseudo‐patients”) varying along several dimensions. Shared dimensions of variation were present in both pseudo‐controls and pseudo‐patients, while specific dimensions of variation were only present in pseudo‐patients. To simulate a control subject, we used a parameterized vector field to either expand or contract a brain by a specified amount (shared effects, Fig. 1, Fig. S1). To simulate a patient, an additional vector field was applied with focal effects to specific brain regions (shared + specific effects). In this way, we created synthetic data with overlapping individual differences in (1) global brain shape that were shared between patients and controls, and (2) individual differences between patients, reflected in varying locations and the magnitudes of disorder‐specific deformations. On average, the shared deformations were 2.4× larger in magnitude than disorder‐specific deformations (Fig. S1). This ratio was chosen to approximate the relative effect sizes of neuroanatomical variations: shared effects (e.g., those associated with age and gender) typically exhibit effect sizes in the range of 0.5–0.8,^15^ whereas disorder‐specific effects (e.g., those linked to schizophrenia, autism, or ADHD) generally have effect sizes ranging from 0.1 to 0.3.^16^, ^17^ In order to test the CVAE across variations in disorder complexity, we generated multiple datasets, with either 2, 3 or 5 subtypes (Datasets 2–4, Table S1, Figs 1, 2). Subtypes were generated by applying disorder‐specific deformations to different locations in the brain (Table S1). Lastly, to investigate disorder‐comorbidity, we generated a dataset with two subtypes, where each subtype had both common and unique disorder loci (Dataset 5, Fig. 3, Table S1). This research was conducted using exclusively synthetic data and, in accordance with Boston College's regulations, was exempt from Institutional Review Board (IRB) approval.

### Vector field parametrization

To control the location and extent of deformations applied to MRI anatomical images, we used parameterized vector fields applied to a template brain image (MNI2009b template^18^). To accomplish this, for each image we: (1) Defined a pair of spheres (reference sphere and a target sphere, Fig. S1) with different radii and with the same center at given coordinates (xyz), (2) Estimated a deformation field from one sphere (the “reference”) to the other (the “target”) using nonlinear registration (implemented in AntsPy^19^) and finally (3) applied this deformation field to the template brain image to generate a new brain image.

### Model architectures and training

The CVAE model followed a previously established architecture^9^ and was based on a modified version of the architecture originally described by Abid & Zou.^8^ Input to the models are 64 × 64 × 64 MRI images, which are then passed through shared and disorder‐specific encoders, each using two convolutional layers, and a decoder with two deconvolutional layers for reconstruction. For controls, reconstruction combines shared features with zeros, while patient data uses both shared and disorder‐specific features (Table S3). Comorbidity‐CVAE uses four encoders to generate latent spaces for shared, patient‐shared, and two disorder‐specific feature spaces (Table S4). A baseline model was a non‐contrastive VAE model, which uses a single encoder with double the filters and a two‐layer decoder (Table S2). All models are optimized using Adam in TensorFlow with a two‐step hierarchical training process—initially training 50 models for 10 epochs, then selecting 20 models with the lowest reconstruction loss to be trained for a full duration of 100‐epoch (for details, see Appendix S1).

### Correlations with ground‐truth effects

In order to establish correlations between the models' latent‐features and ground‐truth effects, we used Representational Similarity Analysis (RSA^20^). For shared features, we calculated pairwise Euclidean distance between subject‐specific latent‐features resulting in a symmetrical matrix quantifying subject similarity according to shared‐features. This procedure was then repeated for the disorder‐specific features. To calculate individual differences in the shared ground‐truth variation, we first calculated the absolute difference in the diameter of the spheres used to produce shared deformations (sphere 1–sphere 2, Fig. S1), resulting in one value per subject specifying the magnitude of shared effects. We then further calculated pairwise differences in the magnitude of shared effects between subjects. We repeated this procedure for the disorder‐specific effects as well. To compare similarity matrices, we extracted upper triangle values for each matrix and correlated them using Kendall's Tau measure. This procedure was repeated for each model in an ensemble and the resulting correlations were averaged. We compared clustering accuracies using a Student's *t*‐test after Fisher Z‐transform. Identical procedure was used for VAE models, using all latent‐features.

### Clustering analyses

To quantify whether CVAE features contain information about neuroanatomical subtypes, we used K‐means clustering. K‐parameter was set according to the ground truth (2,3, or 5 clusters). Training data latent features (shared or disorder‐specific) were concatenated across models within an ensemble (2 features × 20 models). We compared the inferred clustering with ground‐truth subtypes using the Adjusted Rand Index (ARI^21^). ARI values range from −1 to 1, with 1 indicating perfect match and 0 indicating chance. We compared clustering accuracies using a Student's *t*‐test after Fisher Z‐transform. An identical procedure was used for VAE models, using all latent‐features.

### Neurosubtype analyses

In order to identify neural loci associated with each subtype using CVAE, we used the “synthetic‐twin” counterfactual procedure described previously.^8^, ^9^ Each patient's brain was reconstructed twice: once with both shared and disorder‐specific features, and once with only shared features (disorder‐specific set to zero). The latter brain image matches the patient's brain on neuroanatomical features unrelated to the disorder (synthetic twin). We then subtracted the synthetic‐twin brain image from the reconstruction to highlight neuroanatomical loci associated with the presence of the disorder (Fig. 1). For VAE models we followed approaches consistent with those used in the literature.^22^, ^23^, ^24^ This consisted of clustering the neuroanatomical data of patients in the space of VAE latent features, averaging subjects' neuroanatomical images belonging to each cluster to obtain a cluster‐prototype, and subtracting the average control brain from each cluster‐prototype to obtain a map of differences specific to each cluster‐prototype (Fig. 1). To disentangle disease comorbidity, we used the following approach utilizing the comorbidity CVAE model. To identify features associated with disorder 1 in the brains of synthetic patients with disorders 1 and 2, we reconstructed the brains twice: using only shared features (synthetic twin) and again, using shared and disorder 1 specific features, with disorder 2 specific features and comorbid features set to 0. Taking the difference between the two brains highlights the areas associated only with disorder 1. We iterated this procedure to identify brain areas associated with disorder 2 and comorbidity specifically by changing which features were active during the reconstruction.

## Results

### Reliability

While CVAEs have yielded promising results,^9^, ^25^, ^26^ they can be sensitive to weight initializations, which can lead to differences in model fit or model failures (posterior collapse^27^ ^,^ ^28^). To address this, we tested a two‐stage procedure (see Training Procedure) to improve reliability. We compared models' performance on a simulated dataset of *n* = 1000 brains (500 patients, 500 controls; Table S1 Dataset 1). To establish reliability, we repeated training five times using different random seeds. The performance of individual models was not consistent. Collapsing across all model types (CVAEs and VAEs), the average standard deviation was high for variance explained (SD = 0.04), correlations with shared ground truth (SD = 0.03) and correlations with disorder‐specific ground truth (SD = 0.04), see Table S5. As hypothesized, ensemble models were substantially more stable. Averaging within an ensemble of 20 models improved the consistency of the results across five random initializations. The average standard deviation for variance explained was reduced by 4× (SD = 0.01), for correlations with shared ground truth by 2× (SD = 0.015) and for correlations with disorder‐specific ground truth by 5.5× (SD = 0.01), see Table S5.

Similarly, using an ensemble methodology, CVAE shared features correlated reliably with shared ground‐truth measurements (*M* = 0.48, SD = 0.01) and disorder‐specific features correlated reliably with disorder‐specific ground truth (*M* = 0.21, SD = 0.01), Table S5. Furthermore, ensemble models successfully disentangled between shared and disorder‐specific variation (Fig. S2). While it may not be possible to separate shared and disorder‐specific effects completely, disorder‐specific features correlated more strongly with the disorder‐specific ground truth than with the shared ground truth (∆*M* = 0.07, *t*(8) = 9.44, *P*< 0.001). The correlation between shared features and the disorder‐specific ground truth was negligible (*M* = 0.010, SD = 0.001). Shared features correlated more strongly with shared variation than with the disorder‐specific ground truth, *t*(8) = 121.57, *P* < 0.001. Ensemble models converged to similar values for the loss (range: 328–353), including the mean square error (MSE) loss (range: 5.71–6.39) and Kullback–Leibler divergence (KL) loss components (range: 27.85–28.89).

In contrast, correlations between baseline VAE features and ground truth were significantly lower. Disorder‐specific effects were only modestly captured by the VAE model: the correlation between the VAE's features and the ground truth of disorder‐specific variation was *M* = 0.01, compared to the *M* = 0.21 obtained with the CVAE's disorder‐specific features: a 20‐fold difference. This difference was statistically significant (Δ*M* = 0.20, *t*(8) = 30.59, *P* < 0.001). While VAE features did correlate with shared effects (*M* = 0.46), these correlations were also lower when compared to the correlations between the CVAE's shared features and the ground truth of the shared variation (Δ*M* = 0.01, *t*(8) = 3.66, *P* = 0.006). This transpired despite the fact that reconstruction accuracy, in terms of variance explained, was similar between the two models. The difference in variance explained between VAE and CVAE models was not significant (Δ*M* = 0.01, *t*(4) = 1.96, *P* = 0.121).

#### Generalization to independent data

Psychiatric prediction models must generalize beyond training data. However, machine‐learning models trained on one group of subjects often fail to perform when tested on a new group of subjects.^29^ In order to test whether CVAE models can generalize and to avoid issues associated with k‐fold cross‐validation,^29^ we adopted an out‐of‐sample approach. We generated a new dataset (*n* = 1000) with different amounts of shared and disorder‐specific variation sampled from the same distribution. We then used the model trained on one set of subjects to extract latent‐feature representations for an independent group of subjects. Finally, we tested whether correlations with the respective ground‐truth measurements remain high when generalizing to new, unseen data (Fig. S3). Correlations between disorder‐specific features and the ground truth of disorder‐specific variation remained high when tested on independent data (*M* = 0.20, SD = 0.01) and were not significantly different from those obtained when the correlations were calculated using the training data (∆*M* = 0.01, *t*(8) = 1.08, *P* = 0.313). Likewise, the correlation between the shared features and the ground truth of shared variation remained high when using independent data (*M* = 0.48, SD = 0.01) and was similar in magnitude compared to the training data (∆*M* = 0.003, *t*(8) = −0.66, *P* = 0.525). Notably, some standard approaches, like PCA, fail this generalization test. Extracting principal components using the training set (5 PCs, 88% variance explained) results in RSA correlations with disorder specific effects of *r* = 0.02. Performing an analogous generation test, using the loadings trained on one dataset to extract PCs from a different dataset, even when the dataset was sampled from the same distribution, reduces these correlations to 0.004 (5.4× reduction).

### Dataset size

In some situations, data availability might be limited, for example, for rare disorders or due to budget constraints. Therefore, we investigated whether CVAE models are still able to generalize when training data is limited. We used subsamples of the original dataset (*n* = 1000), and trained CVAE with samples of *n* = 500 (250 patients) and *n* = 200 (100 patients). To test generalization, we again extracted latent features for *n* = 1000 independently generated brains and tested correlations with corresponding ground‐truth measurements (Fig. S3). Training on *n*= 500 reduced disorder‐specific correlations compared to *n* = 1000 (∆*M* = 0.04, *t*(8) = 3.14, *P* = 0.014). Correlations between the shared features and the ground truth of shared variation were also attenuated (∆*M* = 0.01, *t*(8) = 3.29, *P* = 0.011). Reducing the training sample size to *n* = 200 had further detrimental effects. Compared to half‐sample (*n* = 500), CVAEs trained with *n* = 200 exhibited lower correlations with both disorder‐specific (∆*M* = 0.09, *t*(8) = 5.53, *P* < 0.001) and shared effects (∆*M* = 0.03, *t*(8) = 10.63, *P* < 0.001). These results indicate that CVAE models are able to generalize given enough training data. With less data, correlations with ground‐truth measurements are progressively lower. Importantly, when using real data, the optimal number of subjects will depend on study‐specific factors, such as the relative amount of shared and disorder‐specific variation in the disorder studied. In addition to dataset size, dataset composition, like the magnitude and ratio of shared and disorder‐specific effects, as well as hyperparameter choices can affect the results (see “hyperparameters” section in the Appendix S1).

### CVAE Subtyping

Identifying disorder subtypes could lead to better diagnosis and development of targeted treatments. Previous subtyping approaches conflate shared and disorder‐specific variation, making it challenging to identify groups of patients with similar disorder‐specific effects. CVAE's disorder‐specific latent space allows for control of disorder‐unrelated (shared) variation that would otherwise confound the results and identify subtypes of patients with similar neuroanatomy by focusing on neural features that are relevant for the disorder. After training the models on a dataset consisting of two neural subtypes (Table S1 Dataset 2), we stacked the latent features of the 20 models within an ensemble and used ARI to evaluate clustering accuracy. For VAE models, we used all features, while for the CVAE, we used only the disorder‐specific features (see methods, Clustering analyses). Compared to the baseline ensemble VAE model (*M* = 0.1), the CVAE ensemble model was significantly more accurate, ∆ADI = 0.69, *t*(8) = 13.08, *P* < 0.001.

Next, we investigated the models' ability to recover patient‐specific neuroanatomical loci. As hypothesized, the clusters discovered by the VAE reflect overall compression/expansion of the brain, which is not specific to patients, but jointly affects controls and patients (Fig. 1, left panel). Correlations between subtypes recovered using VAE and ground truth were low—both on average (*M* = 0.41) and individually (subtype 1 *M* = 0.42, subtype 2 *M* = 0.40). Instead, clusters identified by CVAE were more similar to the ground truth (Fig. 1 right panel). Correlations between CVAE discovered cluster prototypes (Fig. 1 right panel) and the corresponding ground‐truth maps (Fig. 1 middle panel) were high both on average (*M* = 0.87), and individually (subtype 1 *M* = 0.87, subtype 2 *M* = 0.87). These correlations were higher than those discovered by VAE, both on average (∆*M* = 0.46, *t*(8) = 149.83, *P* < 0.001), and when considering subtypes individually (subtype 1: ∆ = 0.45, *t*(8) = 438.48, *P* < 0.001, subtype 2: ∆ = 0.47, *t*(8) = 73.98, *P* < 0.001).

### Multiple subtypes

We next explored the effects of increasing the number of subtypes while using CVAE. Increasing the number of clusters from two to three (Table S1 Dataset 3, Fig. 2) reduced the clustering accuracy to *M* = 0.53 by ∆*M* = 0.26, *t*(8) = 6.54, *P* < 0.001. Increasing clusters from three to five (Dataset 4) further reduced accuracy to *M* = 0.28, ∆*M* = 0.25, *t*(8) = 22.31, *P* < 0.001. We also tested if larger datasets improved clustering accuracy. We doubled the number of subjects (*n* = 2000, 1000 control and 1000 patients) and replicated the analyses. Doubling the number of subjects consistently increased the clustering accuracy for all scenarios: two subtype ∆*M* = 0.11, *t*(8) = 3.98, *P* = 0.004, three subtype ∆*M* = 0.16, *t*(8) = 10.64, *P* < 0.001 and five subtype ∆*M* = 0.29, *t*(8) = 26.68, *P*< 0.001. Similarly, correlations with ground‐truth subtypes had also increased for two‐subtype (∆*M* = 0.06, *t*(8) = 57.97, *P* < 0.001), three‐subtype (∆*M* = 0.003, *t*(8) = 6.04, *P* < 0.001) and five‐subtype cases (∆*M* = 0.08, *t*(8) = 49.51, *P* < 0.001), Fig. 2. To compare these results with subtyping done directly on brain imaging data, we performed PCA on the synthetic neuroanatomical images and retained the minimal number of components that explained >85% of the variance in the data (the number of components retained ranged between 5 and 7 across datasets). Subtyping using neuroimaging data directly resulted in performance at chance level: 2 subtype case ADI = 0.003; 3 subtype case ADI = 0.007; 5 subtype case ADI = 0.0004.

### Comorbidity

Another significant challenge in studying psychiatric heterogeneity is the frequent presence of comorbidities.^30^ Approximately one quarter of patients suffering from a psychiatric disorder also suffer from a comorbid condition.^31^, ^32^ This means that for these patients, we need not only control for variation shared with unaffected controls, but also for variation due to comorbid conditions. To achieve this goal, we used a new synthetic dataset, consisting of disorder subtypes with overlapping neural loci (Table S1 Dataset 5). For convenience, we refer to them as “disorder 1”, “disorder 2” and “disorder 1 + 2”. Each disorder had both shared and unique neural loci (Fig. 4). Applying a modified CVAE architecture successfully disentangled comorbid neural variation. While the input brains had three sources of variation superimposed (disorder 1 specific, disorder 2 specific, and comorbid variation), using the synthetic‐twin approach to suppress variation of non‐interest enabled us to isolate variation selectively associated with disorder 1, disorder 2, or comorbid variation. Importantly, no brains in the training set contained comorbid variation in isolation, as they were always accompanied by shared deformations as well as either disorder 1 or disorder 2 specific deformations. Isolating these effects as shared between disorders demonstrated that CVAE learned to disentangle overlapping sources of variation.

## Discussion

This study demonstrates that Contrastive Variational Autoencoders (CVAEs) are an effective and reliable tool for characterizing psychiatric disorder heterogeneity by disentangling shared and disorder‐specific neural variations, identifying subtypes, and parsing disorder comorbidity. Our results show that CVAEs consistently outperform strong baseline models in separating disorder‐specific effects from broader individual variations, providing a clear advantage in subtype discovery and comorbidity analysis. Using synthetic data with known ground truth allowed for thorough evaluation across varying hyperparameters and dataset sizes, establishing a solid foundation for clinical applications in precision psychiatry, especially with large, complex datasets.

CVAEs have been successfully applied to empirical data in prior studies. Aglinskas *et al*.^9^ showed that autism‐specific neuroanatomical features correlated with symptoms and identified neural loci associated with ASD variability. Zheng *et al*.^13^ identified Parkinson's‐specific features that correlated with clinical severity and could be used to cluster patients into distinct subgroups characterized by either slow or rapid progression of the disease. Kabir *et al*.^10^ identified schizophrenia‐specific features that were related to differences in both symptoms and gene expression profiles. Lastly, Tang *et al*.^14^ used CVAE to identify Alzheimer's‐specific features which were then used to identify brain regions affected by Alzheimer's Disease elucidating potential biological pathways behind its progression. Instead here we focused on synthetic data with known ground truth parameters controlling shared and disorder‐specific variation. This controlled approach allowed for more precise benchmarking, such as evaluating reliability, accuracy and sensitivity to model parameters than would be possible with real‐world datasets. The benchmarks developed in this manuscript lay a solid foundation, informing the analyses of real‐world datasets such as the UK Biobank and ABCD Study.

There are many methods to study disorder‐variability employing linear (e.g. ComBat^33^), probabilistic (e.g. SuStaIn^34^) and deep‐learning (e.g.^35^) models. CVAEs offer key advantages: unlike ComBat, they can control for non‐linear effects between confounds and features of interest; compared to SuStaIn, CVAEs can model variability that is both categorical and continuous (through the activation of model features) whereas SuStaIn focuses on the differences between distinct subtypes. Unlike both ComBat and SuStaIn, CVAEs do not require hand‐selected features, instead discovering relevant patterns in a data‐driven way. By contrast, SuStaIn explicitly models longitudinal trajectories.

Neither ComBat nor SuStaIn can explicitly model disorder comorbidity. In fact, studies investigating comorbid disorders still largely rely on linear methods,^36^, ^37^ while more recent works incorporating machine learning methods place the focus instead on disorder classification.^38^ CVAE approach offers unique advantages for comorbidity studies by isolating latent‐features of neuroanatomy associated with each disorder, as well as their combination. This can be used for downstream tasks, such as predicting clinical variables or identifying affected neural loci *via* synthetic counterfactuals.^9^

Moreover, CVAEs outperform standard deep‐learning models by explicitly controlling for shared *vs*. disorder‐specific variation, de‐confounding disorder‐specific effects from the overshadowing effects of scanner, gender and age. While these approaches are not directly comparable, due to the nature of data they operate on, each has unique advantages and use cases, like longitudinal prediction (SuStaIn), efficient harmonization when covariates are well‐defined (ComBat) and extracting disorder‐specific features in a data‐driven manner (CVAEs).

Here, we specifically analyzed CVAE in the context of neuroanatomy. However, they could potentially be applied to other data modalities as well, such as fMRI,^26^ EEG,^39^ genetic^40^ and behavioral data. Importantly, CVAE approaches could also be used to investigate multimodal data, which is a promising approach for better understanding the biological bases of psychiatric disorders.^41^ Finally, beyond the brain, they could be applied to the imaging of other organs or biological samples.

Neuroimaging biomarkers show promise in predicting symptom progression and response to treatment for a variety of psychiatric conditions, like schizophrenia,^42^ Alzheimer's disease^43^ and depression.^44^ Improving neuromarker validity and reliability using methods like CVAEs could help improve diagnosis and treatment options of psychiatric conditions, which currently affect more than 970 million people.^45^ We recognize the risk that such models might suffer from biases in the datasets that could lead them to generate a more accurate characterization of participants from groups that are overrepresented in the training data. To reduce such biases, it is important to ensure that datasets used to train such models include a diverse population. During data collection, participants should vary in gender, ethnicity, socio‐economic status, and other key dimensions to achieve equitable impact.^46^ During model fitting, incorporating fairness‐aware methods—such as Equality of Opportunity^47^ and counterfactual fairness^48^ can help mitigate model bias. Lastly, during analysis, subgroup analyses^49^ and targeted analyses evaluating model fairness^50^ can help identify and address inequities learned by the models.

### Limitations

As semi‐supervised models, CVAEs require accurate diagnostic labels and can be sensitive to misdiagnosis. While CVAEs are relatively robust to the inclusion of patients in the control dataset (e.g. undiagnosed patients), their performance deteriorates if controls are added to the patients dataset (misdiagnosed controls^8^). In future work, we aim to improve CVAE's robustness to misdiagnosis and to explore novel architectures to relax the semi‐supervised training requirements, for example using sparse autoencoders^51^ or diffusion models.^40^

While CVAEs represent one viable and increasingly popular framework to disentangle disorder‐specific from shared neural features, other generative AI approaches are also emerging with notable promise. For example, Gong *et al*.^52^ review generative models that can create realistic brain images and capture complex network dynamics; Pan *et al*.^53^ use generative adversarial networks for multimodal fusion to identify abnormal circuits in Alzheimer's Disease; Zong *et al*.^54^ leverage graph contrastive learning to highlight disorder‐related connectivity changes in diffusion tensor imaging data; and Le *et al*.,^55^ demonstrate transfer learning or glioma survival prediction. Together, these approaches broaden the toolkit for addressing heterogeneity in brain disorders (also see^56^).

In modeling disorder comorbidity, CVAEs offer a promising tool in capturing comorbid neuroanatomical features in a data‐driven way. Importantly, there will be a need for further research to better understand the complex nature of the relationships between overlapping disorders.

## Author contributions

Conceptualization: A.A., A.B., S.A.; Methodology: A.A, S.A. Formal analysis: A.A.; Funding acquisition: S.A.; Supervision: S.A.; Writing – original draft: A.A., S.A.; Writing – review and editing: A.A., A. B., S.A.

## Disclosure statement

The authors declare that the research was conducted in the absence of any commercial or financial relationships that could be construed as a potential conflict of interest.

## Supporting information


**DATA S1:** Supporting Information.

## Data Availability

Code to generate the data and replicate all analyses is available at: https://github.com/Aglinskas/pub-CVAE-sim-neuroanatomy.
