# Supplementary material for: Understanding heterogeneity in psychiatric disorders: A method for identifying subtypes and parsing comorbidity
Source: Psychiatry Clin Neurosci. 2025 Apr 30;79(7):406–14. doi: 10.1111/pcn.13829 (PMC12232113; doi:10.1111/pcn.13829)
Supplement: Supplementary file 1 — DATA S1: Supporting Information. [file PCN-79-406-s001.docx]

Appendix

**Model architectures**. The CVAE model followed a previously established architecture [(Aglinskas et al., 2022)](https://paperpile.com/c/ndGoij/a1gA) and was based on a modified version of the architecture originally described by [Abid & Zou (2019)](https://paperpile.com/c/ndGoij/Yy85). The CVAE model takes in a batch of anatomical MRI images in 64x64x64 resolution as the input. These images are then passed through two encoders (shared and disorder-specific) and a decoder. The shared encoder employed two successive convolutional layers (kernel size: 3, stride: 2, 64, and 128 convolutional filters). The disorder-specific encoder had an identical structure. These encoders projected the data separately onto parameters of two distinct latent distributions (a distribution on shared features and a distribution on disorder-specific features). The decoder layer took a vector, obtained by concatenating the shared and disorder-specific features, as the input, and produced a reconstructed structural MRI volume as the output. Decoding steps used two deconvolutional layers (kernel size: 3, stride: 2, using 128 and 64 filters), which reconstructed brain images from latent representations. For reconstructing structural volumes of TC participants, the latent representation was a concatenation of the shared-feature representation and a vector of zeros (see [Abid & Zou, 2019](https://paperpile.com/c/ndGoij/Yy85)). The CVAE was trained using batches of 50.

To model comorbid disorders, we modified the base CVAE architecture in the following ways. The comorbidity-CVAE model had four encoders and four sets of latent spaces (4 dimensions each): shared, patient-shared, disorder 1 specific, and disorder 2 specific. TC data was reconstructed using only shared features. Disorder 1 or disorder 2 data was reconstructed using shared, patient-shared, and disorder 1-specific or disorder 2-specific features, respectively. Non-active features were set to zero. Lastly, comorbid disorder 1+2 data was reconstructed using all four sets of features (Table S4, Figure 3).

The baseline model was a traditional convolutional VAE model that was built to be similar to the CVAE, only without the contrastive element. The VAE model had a single encoder which was used to encode both TC and patient brains. It had double the amount of convolutional filters (128, 256, kernel size: 3, stride: 2) to match the total number of filters available to the CVAE (64, 128 per encoder). Similarly, the VAE bottleneck was 4-dimensional and the decoder consisted of two deconvolutional layers (256, 128 filters, kernel size: 3, stride: 2) to reconstruct the original inputs from latent representations. The VAE was trained using batches of 10 to accommodate memory constraints stemming from the larger model size. The VAE model was larger than the comparable CVAE model because of the extra cross-connections needed compared to CVAE (Table S2).

**Training Procedure**. To minimize the effects of random initialization and the risk of posterior collapse [(Dai et al., 2020; Lucas et al., 2019)](https://paperpile.com/c/ndGoij/vy9Y+mbiV) we employed a two-step hierarchical training procedure. In step 1 we trained n=50 models for 10 epochs each. Each epoch consisted of a full pass over the dataset using randomized batches. In step 2 we selected 20 models with the lowest MSE loss to be trained for the whole duration (n=100 epochs). The CVAE training loss was a combination of reconstruction loss (MSE), Kullback-Leibler (KL) divergence loss and a total-correlation loss (see [Abid & Zou; 2019](https://paperpile.com/c/ndGoij/Yy85)). The comorbidity CVAE model training loss was a combination of reconstruction loss (MSE), Kullback-Leibler (KL) divergence loss and three total-correlation losses, penalizing the model for correlations between: 1) shared and all the other latent-features, 2) patient-shared and disorder 1 features and 3) patient-shared and disorder 2 features. The VAE loss was a combination of MSE and KL losses. Model parameters were optimized using the Adam optimizer (learning rate=0.001, beta1=0.9, beta2=0.999, epsilon=1e-07). Models were implemented using TensorFlow 2.3.1, and were trained on an internal cluster with 16GB NVIDIA V100 GPU and 64GB of RAM.

**Computational requirements. For a considerably large dataset of N=1000 subjects, the full ensemble training procedure, which consists of pre-training 50 models for 10 epochs each, then training a selection of 20 models for an additional 100 epochs, performed sequentially on a single NVIDIA V100 GPU with 16GB memory (using batches of 50) takes 14 hours. If additional resources are available, time can be cut down by training models in parallel (in cases of multiple GPUs), or using larger batches (in cases of GPUs with more memory).**

**Hyperparameters**. Deep network models are known to be sensitive to hyperparameters. In order to evaluate this sensitivity for CVAE models, we systematically varied hyperparameters and examined the impact on the results. Increasing latent-space dimensionality of shared and disorder-specific feature space from 2 to 4 units per space resulted in a significant increase in both variance explained and the correlation between disorder-specific features with the corresponding ground-truth measurement. The 4-unit CVAE model explained a larger proportion than 2-unit CVAE (∆ M = 0.03, t(8) = 9.85, p < .001). The correlation between disorder-specific latent space and corresponding ground truth for the 4-unit CVAE was also higher than that of the 2-unit CVAE (∆ M = 0.16, t(8) = 5.59, p < .001), as was the correlation between the shared features correlation and the corresponding ground truth (∆ M = 0.02, t(8) = 5.41, p < .001). Interestingly, increasing dimensionality to 8 units resulted in weaker correlations. Compared to the 4-unit CVAE, the 8-unit CVAE resulted in lower variance explained (∆ M = -0.02, t(8) = -3.11, p = 0.014), lower correlations with shared effects (∆ M = -0.02, t(8) = -5.17, p < .001) and marginally lower correlations with disorder-specific effects (∆ M = -0.07, t(8) = -2.00, p = 0.081). This pattern of effects is likely due to the amount of data (Dataset 1, N=1000, Table S1) used to train all three models being consistent. Optimal network size when working with real data will likely depend on the dataset size, data dimensionality, noise, and other factors. One of the potential ways to optimize network size would be to fit increasingly large models and select the smallest model able to reconstruct 95% of data variance (a similar approach is used to select the optimal number of principal components).

**We next studied how the dataset composition affects the models’ performance. To address this, in separate analyses we varied: A) both disorder-specific and shared effects (keeping the ratio between them constant), and B) shared effects, while keeping disorder-specific effects the same, thus varying the ratio between shared and disorder-specific effects. Even when the ratio between shared and disorder-specific effects remains constant, higher absolute magnitude of the effects led to an increase in correlations between learned disorder-specific features and the corresponding ground truth. At 0.5x the original scale, correlations between disorder-specific features and the corresponding ground-truth were small (M = 0.04), monotonically increasing with larger magnitudes, reaching M = 0.43 when the magnitude of both effects was scaled by 2x, Figure S4A. Similarly, increasing the ratio of disorder-specific to shared effects also affected the resulting correlations with disorder-specific ground truth. When the magnitude of disorder-specific effects was held constant, smaller shared effects (0.5x original magnitude) resulted in higher correlations (M = 0.51), compared to when the disorder-specific effects were entangled with larger shared deformations (2x original magnitude. M = 0.19), Figure S4B. These findings suggest that not only the overall magnitude of the effects, but also their relative ratio, is an important consideration for CVAE applications.**

**Then, we varied the β hyperparameter (the KL‐divergence scaling) from 0.1× to 10×. We again observed a monotonic increase in correlations with ground truth, ranging from M = .16 at 0.1x scaling, to M = .26 at 10x, Figure S4C. Lastly, when we varied the γ hyperparameter used to penalize shared information between latent spaces, we observed a deterioration in models’ performance when this parameter was scaled to either 0.1x (M = 0.21) or 10x (M = .16), Figure S4D. These findings suggest that while the default hyperparameter values provide a solid baseline performance, refining both model-specific factors (bottleneck dimensionality, β, γ) and dataset characteristics (proportion of shared vs. disorder-specific effects) can affect model performance. While currently there is no one gold-standard way to optimize hyperparameters, literature is converging on best practices (Bisch et al., 2023) and developing automated tools for hyperparameter selection (Luo, 2016).**

References

[Aglinskas, A., Hartshorne, J. K., & Anzellotti, S. (2022). Contrastive machine learning reveals the structure of neuroanatomical variation within autism. *Science*, *376*(6597), 1070–1074.](http://paperpile.com/b/ndGoij/a1gA)

[Abid, A., & Zou, J. (2019). Contrastive variational autoencoder enhances salient features. *arXiv Preprint arXiv:1902. 04601*.](http://paperpile.com/b/ndGoij/Yy85)

[Dai, B., Wang, Z., & Wipf, D. (2020). The usual suspects? Reassessing blame for VAE posterior collapse. *International Conference on Machine Learning*, 2313–2322.](http://paperpile.com/b/ndGoij/mbiV)

[Lucas, J., Tucker, G., Grosse, R., & Norouzi, M. (2019). Don’t blame the ELBO! A linear VAE perspective on posterior collapse. *Advances in Neural Information Processing Systems*, *abs/1911.02469*.](http://paperpile.com/b/ndGoij/vy9Y) <https://proceedings.neurips.cc/paper/2019/hash/7e3315fe390974fcf25e44a9445bd821-Abstract.html>

**Bischl, B., Binder, M., Lang, M., Pielok, T., Richter, J., Coors, S., ... & Lindauer, M. (2023). Hyperparameter optimization: Foundations, algorithms, best practices, and open challenges. Wiley Interdisciplinary Reviews: Data Mining and Knowledge Discovery, 13(2), e1484.**

**Luo, G. (2016). A review of automatic selection methods for machine learning algorithms and hyper-parameter values. Network Modeling Analysis in Health Informatics and Bioinformatics, 5, 1-16.**


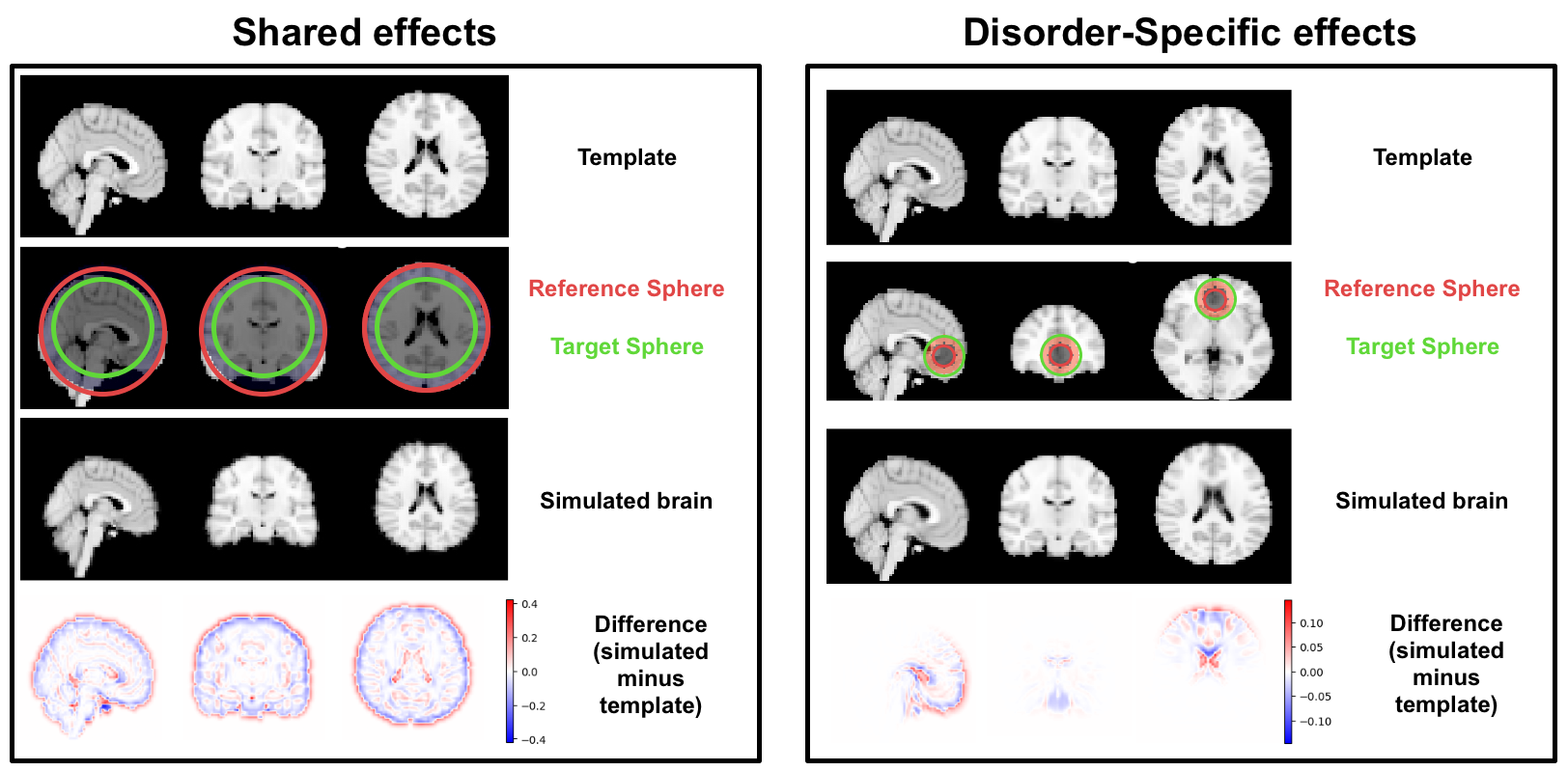


Fig. S1: Generating simulated brains with shared and specific variation effects. a) Shared effects were exemplified by global brain expansion/compression. b) Disorder-specific effects were exemplified by focal expansion/compression, for example, in the frontal lobe. To quantify the relative magnitude of shared versus disorder-related transformations, we used Jacobian determinant maps. Specifically, we calculated the Jacobian determinant for each vector field specifying a deformation (shared or specific). This results in a value specifying the relative compression (values less than 1) or expansion (values more than 1) for each voxel. We calculated the absolute value after subtracting 1 from the Jacobian maps. Then, we summed across voxels to obtain a single value, each representing the magnitude of shared or specific deformations applied. The ratio between the two indicated the shared deformations were, on average, 2.4x larger than disorder-specific deformations.


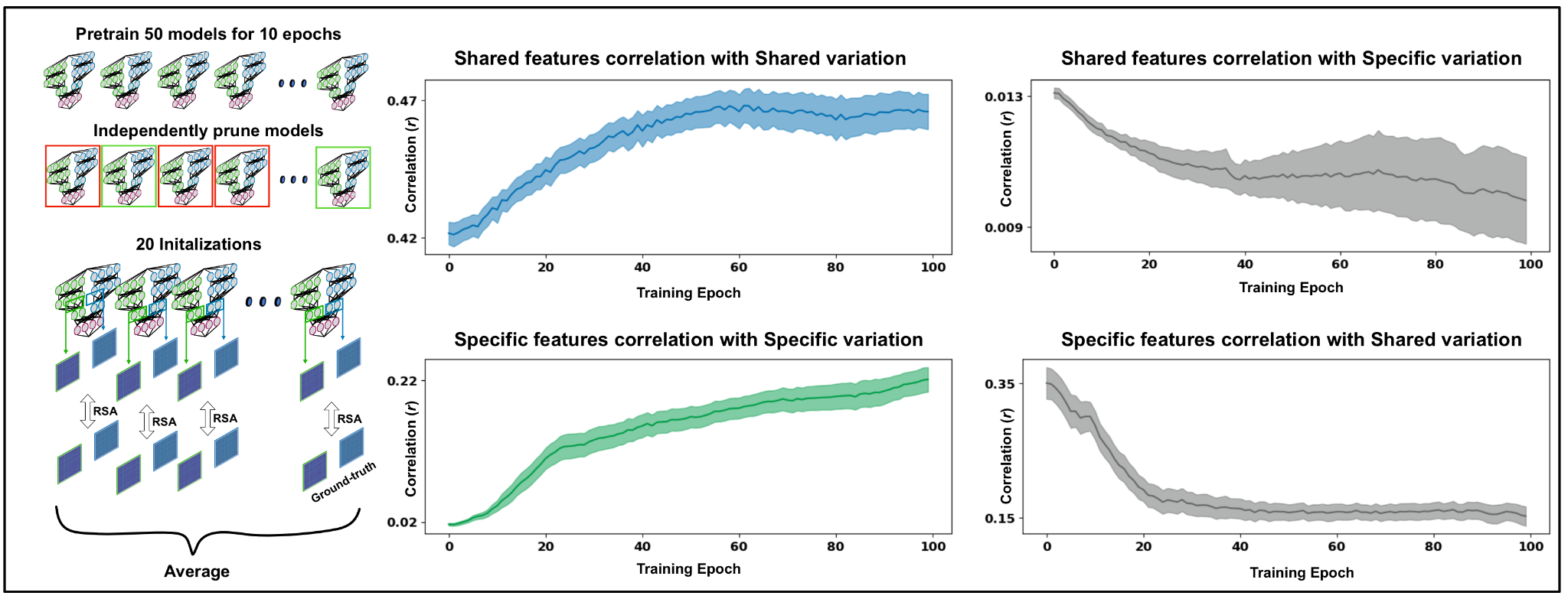


Fig. S2: CVAE reliability using model ensembling. Line shows average correlation between latent features and ground-truth measurements across 20 different initializations; the shaded bars show SEM. Correlations with shared (blue) and specific (green) ground-truth measurements show steady increase during training. Correlations between shared features and specific ground truth (and vice-versa) instead get increasingly lower as training progresses, indicating successful disentangling of shared and specific features.


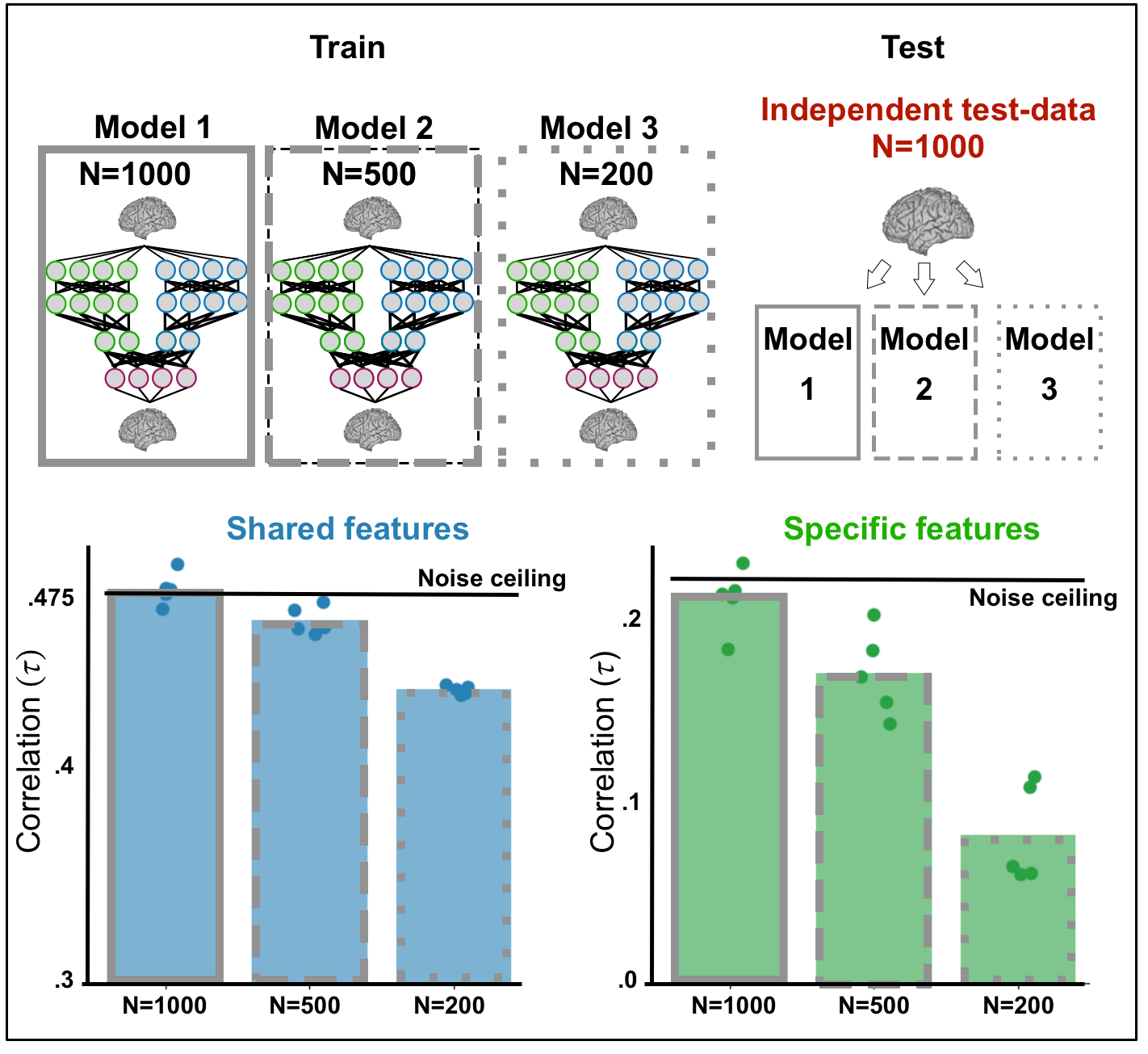


Fig. S3: CVAE generalization. We trained separate CVAE models using three different training set sizes (N=1000, N=500, and N=200). Then we tested each model using independent data (N=1000) not used during training. CVAE models trained with N=1000 subjects show high correlations with ground-truth measurements even when tested with independent data (at noise ceiling). These correlations get progressively weaker when training data is limited. Noise ceiling was calculated by extracting shared and disorder-specific features using training data and correlating them to corresponding ground-truth measurements. **The** **noise ceiling line represents the models’ performance when trained and tested on the same dataset, while the height of the bars shows generalization performance, when a model is trained on one dataset and features are extracted from a test dataset from the same distribution (see section “Generalization to independent data” in the main text).**

**
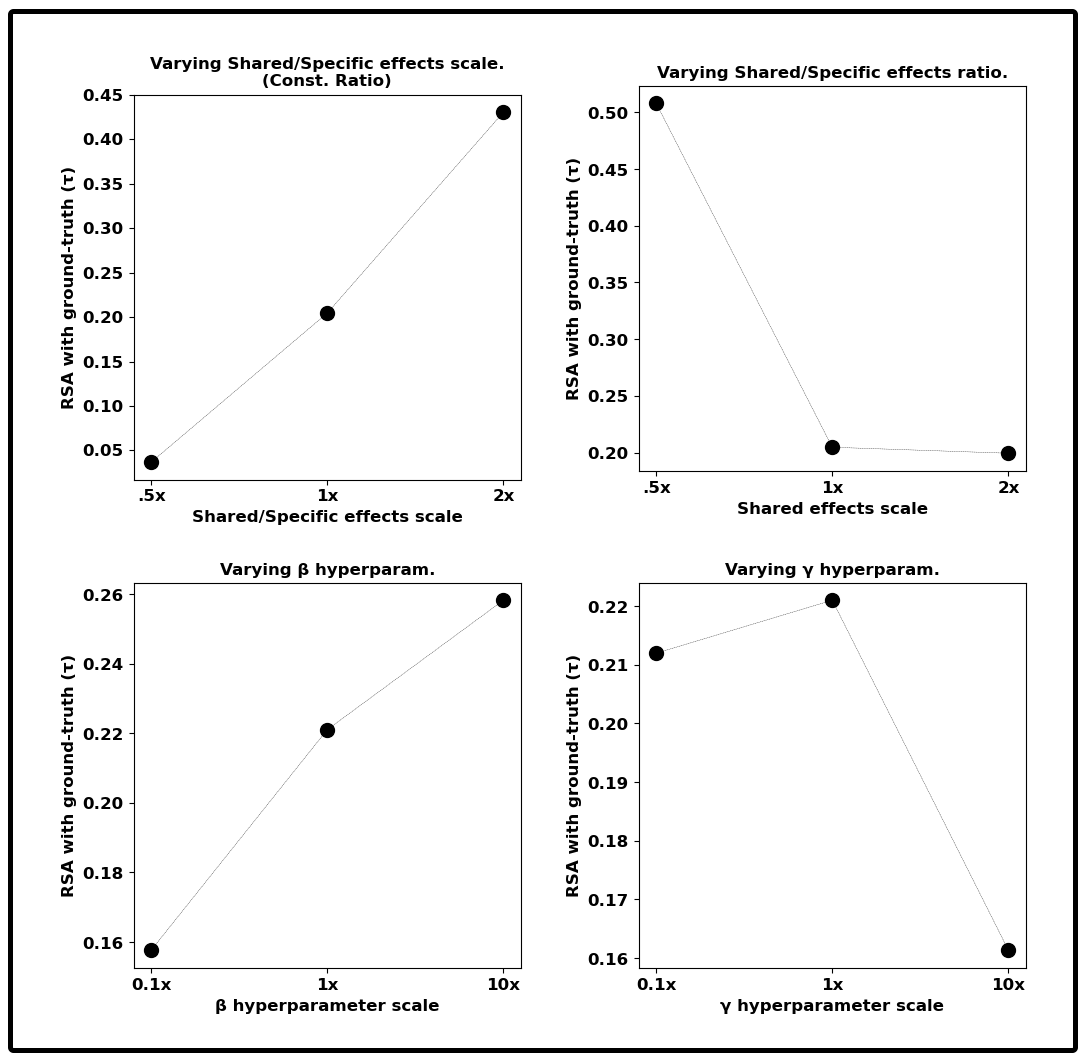
**

**Fig S4: Effects of varying model and data hyperparameters. A) Increasing both the shared and disorder-specific effects magnitude simultaneously (keep the ratio between them constant) results in higher correlations between disorder-specific features and the corresponding ground truth. B) If the disorder-specific effect magnitude is constant, larger shared variations result in lower correlations between disorder-specific features and ground truth. C) Increasing the β hyperparameter, which scales the KL divergence term in VAEs, increases correlations with ground-truth. D) Increasing the hyperparameter γ, which scales the penalty for correlations between latent spaces, has a clear optimum, with performance drops if this value is increased or decreased.**

Table S1: List and description of datasets used.

| Dataset name | Shared effects  coordinates | Specific effects  Coordinates (MNI) | Number of subtypes | Number of subjects |
| --- | --- | --- | --- | --- |
| Dataset 1 | MNI: -1,-20,17 | -1,35,-3 | N/A | 1000 |
| Dataset 2 | MNI: -1,-20,17 | -1,35,-3  -1,-54,31 | 2 | 1000 / 2000 |
| Dataset 3 | MNI: -1,-20,17 | -1,35,-3  -1,-54,31  -1,-61,23 | 3 | 1000 / 2000 |
| Dataset 4 | MNI: -1,-20,17 | -1,35,-3  -1,-54,31  -1,28,42  -1,-61,-23  4,8,8 | 5 | 1000 / 2000 |
| Dataset 5 | MNI: -1,-20,17 | -53,-13,-18  -1,35,-3  -1,-54,31 | 3 | 2000 |

Table S2. Architecture of the VAE model.

| Layer Name | Output Shape | # Params |
| --- | --- | --- |
| Encoder | | |
| InputLayer | [(None, 64, 64, 64, 1)] | 0 |
| conv3d 1 (Conv3D) | (None, 32, 32, 32, 128) | 3584 |
| conv3d 2 (Conv3D) | (None, 16, 16, 16, 256) | 884992 |
| flatten (Flatten) | (None, 1048576) | 0 |
| dense (Dense) | (None, 128) | 134217856 |
| z_mean (Dense) | (None, 4) | 516 |
| z_log_var (Dense) | (None, 4) | 516 |
| Decoder | | |
| z_sampling (InputLayer) | [(None, 4)] | 0 |
| dense (Dense) | (None, 128) | 640 |
| dense (Dense) | (None, 1048576) | 135266304 |
| reshape (Reshape) | (None, 16, 16, 16, 256) | 0 |
| conv3d_transpose 1 (Conv3DTranspose) | (None, 32, 32, 32, 256) | 1769728 |
| conv3d_transpose 2 (Conv3DTranspose) | (None, 64, 64, 64, 128) | 884864 |
| decoder_output (Conv3DTranspose) | (None, 64, 64, 64, 1) | 3457 |
| Total # params: 273,032,457 | | |

Table S3. Architecture of the CVAE model.

| Layer Name | Output Shape | # Params |
| --- | --- | --- |
| Disorder-Specific Encoder | | |
| InputLayer | (None, 64, 64, 64, 1) | 0 |
| conv3d 1 | (None, 32, 32, 32, 64) | 1792 |
| conv3d 2 | (None, 16, 16, 16, 128) | 221312 |
| flatten (Flatten) | (None, 524288) | 0 |
| dense (Dense) | (None, 128) | 67108992 |
| z_mean (Dense) | (None, 2) | 258 |
| z_log_var (Dense) | (None, 2) | 258 |
| Shared Encoder | | |
| InputLayer | (None, 64, 64, 64, 1) | 0 |
| conv3d 1 | (None, 32, 32, 32, 64) | 1792 |
| conv3d 2 | (None, 16, 16, 16, 128) | 221312 |
| flatten (Flatten) | (None, 524288) | 0 |
| dense (Dense) | (None, 128) | 67108992 |
| z_mean (Dense) | (None, 2) | 258 |
| z_log_var (Dense) | (None, 2) | 258 |
| Decoder | | |
| z_sampling | [(None, 4)] | 0 |
| dense (Dense) | (None, 128) | 640 |
| dense (Dense) | (None, 524288) | 67633152 |
| reshape (Reshape) | (None, 16, 16, 16, 128) | 0 |
| conv3d_transpose 1 (Conv3DTranspose) | (None, 32, 32, 32, 32) | 110624 |
| conv3d_transpose 2 (Conv3DTranspose) | (None, 64, 64, 64, 16) | 13840 |
| decoder_output (Conv3DTranspose) | (None, 64, 64, 64, 1) | 433 |
| Total params: 202,423,918 | | |

Table S4. Architecture of the comorbidity CVAE model.

| Layer Name | Output Shape | # Params |
| --- | --- | --- |
| Encoder (x4) | | |
| InputLayer | (None, 64, 64, 64, 1) | 0 |
| conv3d 1 | (None, 32, 32, 32, 64) | 1792 |
| conv3d 2 | (None, 16, 16, 16, 128) | 221312 |
| flatten (Flatten) | (None, 524288) | 0 |
| dense (Dense) | (None, 128) | 67108992 |
| z_mean (Dense) | (None, 2) | 516 |
| z_log_var (Dense) | (None, 2) | 516 |
| Decoder | | |
| z_sampling | [(None, 16)] | 0 |
| dense (Dense) | (None, 128) | 2176 |
| dense (Dense) | (None, 524288) | 67633152 |
| reshape (Reshape) | (None, 16, 16, 16, 128) | 0 |
| conv3d_transpose 1 (Conv3DTranspose) | (None, 32, 32, 32, 32) | 110624 |
| conv3d_transpose 2 (Conv3DTranspose) | (None, 64, 64, 64, 16) | 13840 |
| decoder_output (Conv3DTranspose) | (None, 64, 64, 64, 1) | 433 |
| Total params: 202,872,752 | | |

Table S5. Effects of model ensembling. Variance explained and correlation values before and after model ensembling.

| Architecture | M | SD | Range |
| --- | --- | --- | --- |
| Variance explained: individual models | | | |
| VAE | 0.88 | 0.06 | 0.63-0.95 |
| CVAE | 0.89 | 0.02 | 0.80-0.92 |
| Variance explained: ensemble models | | | |
| VAE | 0.88 | 0.01 | 0.85-0.89 |
| CVAE | 0.89 | 0.01 | 0.88-0.90 |
| Correlations with shared ground truth: individual models | | | |
| VAE | 0.46 | 0.03 | 0.41-0.52 |
| CVAE | 0.48 | 0.03 | 0.41-0.53 |
| Correlations with shared ground truth: ensemble models | | | |
| VAE | 0.46 | 0.01 | 0.46-0.47 |
| CVAE | 0.48 | 0.02 | 0.47-0.49 |
| Correlations with disorder-specific ground truth: individual models | | | |
| VAE | 0.010 | 0.008 | 0.000-0.033 |
| CVAE | 0.214 | 0.069 | 0.051-0.379 |
| Correlations with disorder-specific ground truth: ensemble models | | | |
| VAE | 0.010 | 0.001 | 0.009-0.012 |
| CVAE | 0.214 | 0.013 | 0.191-0.230 |
